# Antioxidant Generation during Coffee Roasting: A Comparison and Interpretation from Three Complementary Assays

**DOI:** 10.3390/foods3040586

**Published:** 2014-11-12

**Authors:** Sebastian E. W. Opitz, Samo Smrke, Bernard A. Goodman, Marco Keller, Stefan Schenker, Chahan Yeretzian

**Affiliations:** 1 Institute of Chemistry and Biological Chemistry, Zurich University of Applied Sciences, Einsiedlerstrasse 31, CH-8820 Wädenswil, Switzerland; E-Mails: opit@zhaw.ch (S.E.W.O.); opit@zhaw.ch (S.S.); 2 State Key Laboratory for Conservation and Utilization of Subtropical Agro-Bioresources, Guangxi University, Nanning, 530004 Guangxi, China; 3 Bühler AG, Gupfenstrasse 5, 9240 Uzwil, Switzerland; E-Mails: marco.keller@buhlergroup.com (M.K.); stefan.schenker@buhlergroup.com (S.S.)

**Keywords:** coffee, antioxidant assays, Folin-Ciocalteu, ABTS, ORAC, flow injection analysis

## Abstract

Coffee is a major source of dietary antioxidants; some are present in the green bean, whereas others are generated during roasting. However, there is no single accepted analytical method for their routine determination. This paper describes the adaption of three complementary assays (Folin-Ciocalteu (FC), ABTS and ORAC) for the routine assessment of antioxidant capacity of beverages, their validation, and use for determining the antioxidant capacities of extracts from coffee beans at different stages in the roasting process. All assays showed a progressive increase in antioxidant capacity during roasting to a light roast state, consistent with the production of melanoidins having a higher antioxidant effect than the degradation of CGAs. However, the three assays gave different numbers for the total antioxidant capacity of green beans relative to gallic acid (GA), although the range of values was much smaller when chlorogenic acid (CGA) was used as reference. Therefore, although all three assays indicated that there was an increase in antioxidant activity during coffee roasting, and the large differences in responses to GA and CGA illustrate their different sensitivities to different types of antioxidant molecule.

## 1. Introduction

Epidemiological studies have revealed that coffee consumption is linked to several beneficial health effects [1,2]. Furthermore, it has been suggested that these are associated with the high antioxidant capacity of the beverage, which is greater than in most other food products [3,4,5,6], and represents a major source of antioxidants in the diets of many people [7,8,9].

In many plant organs, phenolic compounds are the most abundant antioxidant molecules and the phenolic content is often considered to represent a good approximation of the total antioxidant capacities of foods and beverages [10,11]. Thus, beneficial health effects have been strongly linked to (poly)phenolic components, and in tea, for example, flavonoids account for much of the antioxidant capacity [12], and have been specifically linked to its health-related properties [13].

In unroasted coffee beans, the chlorogenic acids (CGAs) are a major source of the total antioxidant capacity [14,15] with a high level of bioavailability [16], although as esters of primarily caffeic and quinic acids, they belong to a different sub-family of phenols from those in teas. Also, CGAs are at least partially degraded during the roasting process [17,18], which also results in the production of different types of antioxidant molecules, most notably the ill-defined products from the Maillard and caramelization reactions [19,20]. The water-soluble, macromolecular products from these reactions, known as melanoidins, have been linked to many chemical and biological properties, and are responsible for the taste and color of coffee [20]. Thus with this beverage, measurements of polyphenol contents are unlikely to give a complete picture of its antioxidant properties.

A wide range of assays have been reported for the measurement of antioxidant capacities of food products [1,2,3,4,5,6,7,8,9,10,11,12,13,14,15,16,17,18,19,20,21,22,23,24,25], but many are based on different types of chemical reaction and they do not all provide equivalent information. There are also many reports of the antioxidant capacity of coffee, in particular its dependence on the roast degree, but different assays have been used, and there are inconsistencies between results reported by different groups [17,18,19,26,27,28,29,30,31]; for example, conditions reported for the highest antioxidant potential have varied over a wide range of roasting conditions from green coffee through light to medium to dark roasted coffee, and the results are seemingly dependent on the method used. Thus, in order to get an accurate description of the antioxidant properties of coffee brews, and to produce information that may be relevant to the health effects of coffee consumption, it is necessary to have an understanding of the chemical basis of the various assays, which should be able to detect both phenolic and non-phenolic antioxidant molecules. In addition, it is important that assays and their experimental protocols are widely accepted in order to compare results obtained in different studies. Finally, although cellular assays [32] can provide biological support for any chemical findings, ultimately health properties can only be firmly established by true *in vivo* studies, which are subject to many confounding factors. Thus at the present time we are forced to rely to a large extent on chemical assays for understanding the antioxidant properties of food products.

A major aim of our current research program is to develop an analytical platform for assessing the antioxidant properties of coffee brews that is both easy to operate and can generate highly reproducible results, whilst at the same time providing values that are relevant to quality perception and biological properties of the beverage [33]. In the present paper, we describe the determination of antioxidant properties of brews prepared from green coffee beans and beans roasted at three different speeds and to different temperatures in an attempt to understand the critical stages in the roasting process for the formation and destruction of antioxidant molecules. In order to do this we have adapted the experimental protocols for three commonly-used complementary assays for determining the antioxidant capacity of food products to the routine and easy assessment of the antioxidant properties of coffee using automated flow injection techniques. Two of these assays are based on electron transfer reactions, namely the Folin-Ciocalteu (FC) assay for total phenols [34], and the ABTS assay, which is based on oxidation of the reagent to the cationic chromophore, 2,2’-azinobis-(3-ethylbenzothiazoline-6-sulphonate) (ABTS^•+^) [35]. The third assay, which is based on hydrogen atom transfer, is the oxygen radical absorbance capacity (ORAC), in which the antioxidant and the substrate fluorescein compete for thermally generated radicals after their formation by decomposition of 2,2’-azobis-2-amidinopropane, dihydrochloride (AAPH) [36]. Therefore, in addition to presenting their applications to the measurement of antioxidant properties of extracts of coffee at various stages of the roasting process, we also describe the optimization of these analytical methods for reproducibility, accuracy and speed of analysis and critically assess the information that is produced by them.

## 2. Experimental Section

### 2.1. Materials

Gallic acid monohydrate (purity > 99%) (GA), 2,2’-azobis-2-amidinopropane-dihydrochloride (AAPH), 2,2’-azinobis-(3-ethylbenzothiazoline-6-sulphonate) (ABTS), Folin-Ciocalteu reagent, salts and solvents were purchased from Sigma-Aldrich (Buchs SG, Switzerland). 5-Caffeoyl quinic acid (5-CQA) was obtained from Acros Organics (Geel, Belgium).

### 2.2. Coffee Roasting and Preparation

All coffee measurements were performed on a single batch of *Coffea arabica* L. beans from Costa Rica (washed Costa Rica, strictly hard bean). Coffee samples were roasted in a 20 kg batch roaster (Roastmaster™ 20, Bühler AG, Uzwil, Switzerland) according to three profiles, described as fast, medium and slow (Table 1). Within these profiles, each sample was prepared separately by roasting 8 kg of green coffee beans to a certain temperature along the profile. Beans were roasted and removed at the following temperatures: (1) 150, 170, 190, 203, 209 and 215 °C for fast, (2) 130, 150, 170, 190, 203, 209 and 215 °C for medium, and (3) 110, 130, 150, 170, 190, 203, 209 and 215 and 220 °C for slow roasting conditions. The lower roasting temperatures were performed twice, whereas the higher roasting temperatures (209, 215, and 220 °C) were only performed once. After a storage period of a minimum of 10 days in a refrigerator at 4 °C, the coffee samples were ground with a ball mill (MM 400, Retsch GmbH, Haan, Germany) for 1 min at 30 Hz. For green coffee beans, as well as those that were only partially roasted (to 110 or 130 °C), the samples were cooled in liquid nitrogen before grinding in order to obtain finely ground powders. The mass loss and the humidity content were determined by gravimetric analysis, the latter as loss of water in % after roast and ground (R & G) coffee was dried at 120 °C for 10 min with a Halogen Moisture Analyzer (HG 63, Mettler Toledo, Greifensee, Switzerland) (Table 1). Finally, the color of the roasted and ground coffee was determined with a Colortest II (NeuhausNeotec GmbH, Reinbek, Germany), which showed that the samples removed at 203 °C corresponded to a light roast. For analysis, the coffee samples were brewed in triplicate using a 200 mL French press (Bodum, Triengen, Switzerland) in which 12 g of ground coffee powder was infused with approximately 200 mL (mean value 190.5 ± 0.5 g) of hot water at 92 °C [18].

**Table 1:** Summary of roasting data for Arabica coffee samples for different profiles and roasting degrees (*n* = 1)

Roast profile	Final roast temperature °C	Roast time min:sec	Color of R & G coffee Colortest II	Mass loss %	Humidity %
Fast	150	03:00 ± 4	166.8 ± 0.7	5.8	ND
	170	03:52 ± 5	154.0 ± 1.0	8.7	4.17
	190	04:43 ± 2	130.2 ± 7.78	12.0	3.32
	203	05:15 ± 0	97.2 ± 13.44	14.7	2.26
	209	05:27	71.3	16.4	2.19
	215	05:37	56.3	18.6	1.84
Medium	130	03:50 ± 6	ND	4.8	ND
	150	05:07 ± 10	161.5 ± 2.1	7.0	ND
	170	06:45 ± 2	160.7 ± 1.9	9.4	4.06
	190	08:23 ± 19	139.5 ± 3.1	12.3	3.16
	203	09:36 ± 21	109.4 ± 6.6	15.3	2.03
	209	10:29	93.7	15.9	1.43
	215	11:05	78.7	17.6	1.26
Slow	110	03:07 ± 17	ND	2.8	ND
	130	04:54 ± 9	ND	5.0	ND
	150	06:45 ± 2	162.3 ± 1.4	7.4	ND
	170	09:06 ± 1	162.5 ± 0.2	9.5	4.16
	190	12:04 ± 4	140.7 ± 0.0	12.6	3.06
	203	14:51 ± 24	106.8 ± 2.1	15.3	1.92
	209	15:48	91.7	15.9	1.74
	215	16:36	78.0	16.8	1.32
	220	17:51	67.3	19.2	1.19

The coffee was roasted either at a fast, a medium or a slow roast profile and each coffee for a given roast temperature was roasted separately. Beans were removed at the presented temperatures, which correspond to the roasting time. Mass loss, humidity and color of the ground coffee were measured, where applicable. ND = not determined.

### 2.3. Antioxidant Assays

Each sample was analyzed by each of the three following assays that are commonly used for assessment of antioxidant values. In each case, the coffee extracts were diluted to adjust the concentration to the specific dynamic range of the assays. Samples for the FC assay were diluted 1:50, for the ABTS assay 1:200, and 1:400 for the ORAC assay, and analyzed with a FIA lab 3200 instrument (FiALab Instruments Inc., Bellevue, WA, USA). A Cetac ASX-260 autosampler (Omaha, NE, USA) together with a 1 mL syringe pump were used for automatic introduction of the samples for all assays. In the FC assay the antioxidant capacity is measured with a flow injection analysis (FIA) setup including a six-port injection valve. Both FC reagent and NaOH solutions are pumped simultaneously and the sample/standard is injected with the six-port valve, which is filled by the autosampler. Constantly pumped FC reagent carries the sample from the injection loop towards the T-piece, where the solution mixes with the NaOH solution. After further mixing and dispersion in the reaction coil, the absorbance of the blue colored complex product is measured in the detector cell. In the ABTS assay the antioxidant capacity is measured via a sequential injection analysis (SIA) setup. After the transfer tube from the autosampler is purged with sample/standard, the carrier syringe is pre-filled with carrier solution, followed by sequential stacking of reagents (ABTS^•+^, sample and carrier) in the holding tube. By delaying subsequent actions for 30 s, the reaction time of the stacked reagents is prolonged before the ABTS^•+^ absorbance is measured in the detector cell. In the ORAC assay the antioxidant capacity is measured via a sequential injection analysis (SIA) setup using a stop flow technique in the detector cell. After the transfer tube from the autosampler is purged with sample/standard, the carrier syringe is pre-filled with carrier solution, followed by sequential stacking of reagents (AAPH, fluorescein, sample and carrier) in the holding tube. Then the content of the holding coil is dispensed through the detector cell, stopped at the peak of the fluorescein signal and the degradation of fluorescein is monitored for 1 h.

All samples were filtered before analysis using 0.45 μm polyethylene terephthalate filters (Machery-Nagel, Düren, Germany). Results are expressed as gallic acid equivalents (GAE), which were used as a standard reference material; this was preferred to the more commonly-used Trolox, because of solubility problems of the latter in our system.

#### 2.3.1. Folin-Ciocalteu (FC) Assay

**Figure 1:**
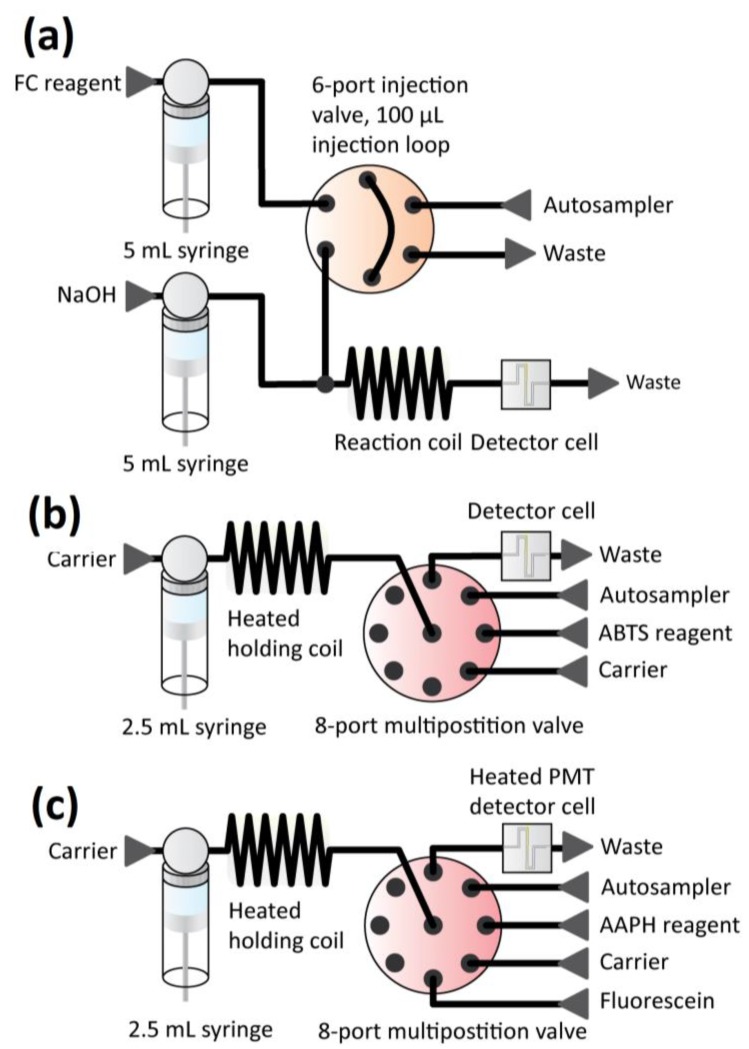
Schemes for antioxidant measurements by (**a**) FC, (**b**) ABTS and (**c**) ORAC assays.

This assay makes use of the fact that phenolic compounds undergo autoxidation in an alkaline medium, and that these products then react with the FC reagent, which consists of a mixture of phosphomolybdate and phosphotungstate, to form “molybdenum blue” [34], which is then measured. Our automated adaption of the FC assay consists of a flow injection analysis (FIA) setup, in which the sample or antioxidant standard is injected (injection loop 100 μL) into the flow stream (flow rate 30 μL/s) of the FC reagent (0.2 M with respect to acid) (Figure 1a). After mixing with NaOH solution (0.25 M, flow rate 30 μL/s) to raise the solution pH (to ~pH 10), dispersion in the reaction coil (1 m tubing length) mixes the components, and the molybdenum blue reaction product is measured photometrically at 765 nm.

#### 2.3.2. ABTS (2,2’-Azinobis-(3-Ethylbenzothiazoline-6-Sulphonate)) Assay

In the ABTS assay, which is also known as the Trolox equivalent antioxidant capacity (TEAC) assay, the green-blue stable radical cationic chromophore, ABTS^•+ ^ is reduced by antioxidants and a subsequent drop in color intensity is observed. The radical cation has to be generated prior to analysis, because it is not sufficiently stable to be available in bulk. Several different approaches have been published for ABTS^•+^ generation and in the present work we used oxidation by potassium persulfate as originally described by Re *et al.* [35]. The assay was performed using a sequential injection analysis (SIA) setup to minimize consumption of the reagent (Figure 1b). The sample or antioxidant standard (injection volume 40 μL), ABTS^•+^solution (injection volume 20 μL) and carrier solution (0.1 M Na_2_HPO_4_, pH = 7.4) were aspirated sequentially into a heated holding coil, which was thermostated at 35 °C. By aspirating and dispensing the components through the holding coil, an even dispersion was achieved; the instrument was stopped after the sampling process was completed to achieve a total reaction time of 60 s [35,37]. After dispensing the mixture to the detector cell (flow rate 30 μL/s), the absorbance of the ABTS^•+^ solution was measured photometrically at 734 nm. Before starting the experiment, the concentration of ABTS^•+^ solution was set to an initial absorbance of 0.75 AU as the preparation of the ABTS^•+^ solution was not accurately reproducible.

#### 2.3.3. Oxygen Radical Absorbance Capacity (ORAC) Assay

The ORAC assay measures the effect of a test sample on the decrease in fluorescence of fluorescein under a constant flux of free radicals that originate from AAPH and result in the formation of a non-fluorescent product [36]. When antioxidants are present, a proportion of the radicals reacts directly with an antioxidant molecule abstracting a hydrogen atom to form a diamagnetic product and a “stable” antioxidant-derived radical, which does not react with fluorescein. However, although a proportion of the reactive radicals still reacts with fluorescein, there is a secondary reaction between antioxidants and fluorescein radicals that results in the regeneration of fluorescein. This latter reaction results in a lag phase in the fluorescein degradation [38], and the results from the assay represent a combination of both types of reaction. By quantifying the integrated area under the curve of the fluorescence decay, this assay combines several ways of quantification of antioxidant capacity, e.g., lag time, initial rate, and total inhibition in a single value [22]. Our adaption of the original assay [36] uses a SIA setup in which the sample or antioxidant standard (injection volume of 25 μL), fluorescein solution (25 μL of 0.37 μg/mL solution), and the AAPH solution (50 μL of 50 mg/mL solution) are aspirated sequentially into a holding coil, which is thermostated at 35 °C (Figure 1c). However, the incubation step at 37 °C described by Ou *et al*. [36] was omitted from the AAPH preparation procedure. A crucial step in our assay is the control of the initial formation of radicals from AAPH. Therefore, the AAPH was stored in ice and the tubing purged each time with cold AAPH before use. Introducing the components and the carrier (0.1 M Na_2_HPO_4_, pH 7.4) into the holding coil produced an even dispersion in a buffered solution, as the fluorescence signal of fluorescein is pH dependent [36]. Subsequently the reagents-sample mixture was dispensed to the detector cell, which was also thermostated at 35 °C. At the peak of the fluorescein signal, the flow was stopped and degradation of fluorescein was monitored for 1 h in a photomultiplier tube. A 5 mm light emission diode combined with a blue color filter was used as an excitation source and an interference green filter was placed before the photomultiplier tube to filter the emitted light. Area under the curve (AUC)-quantification was then applied to the fluorescein decay curve to produce a measure of the antioxidant value.

### 2.4. Validation of Assays

Each assay was validated before being used in routine measurements of coffee antioxidant capacity. The validation was performed with GA as the reference compound for each assay, and consisted of determinations of repeatability within a day (intraday), reproducibility between days (interday), the limit of detection (LoD), limit of quantification (LoQ), linearity range, coefficient of determination and recovery percentage.

After preparation of a GA stock solution in degassed water, working standard solutions were prepared at appropriate concentrations for the linearity range of the assays. Intraday repeatability was assessed by repetitive injections of a single standard GA sample. Interday reproducibility was performed over a period of several days and involved the repeated preparation of a calibration curve of freshly prepared standards and reagents. The LoD and LoQ were investigated by using GA standard solutions at low concentrations, and the dynamic range for the analyses was determined from the linearity of the calibration curve and the LoD. Assessment of recovery was made by spiking samples of a freeze-dried soluble coffee (dissolved in 100 mL of degassed water) with a GA standard solution.

In addition to these standard validation criteria, robustness tests were carried out on certain parameters of some assays. Because the ABTS^•+^ reagent degrades slowly, the stability of the ABTS^•+^ radical is a critical factor for accurate measurements, and this was tested by injecting a blank sample and a 3 mg/L GA standard every hour during measurements. Further, the initial ABTS^•+^ concentrations were varied to investigate the robustness of the initial absorbance. Also, although results obtained by the ABTS assay can be expressed either as an absorbance decrease, which is an absolute difference in absorbance between a blank and a sample signal, or as an inhibition percentage (Inh. %), we only present data for the latter, which represents the ratio between the observed absorbance after the reaction with an antioxidant and that of the maximum value of the blank (water).

For the ORAC assay, AAPH and fluorescein concentrations were varied to test the response of selected concentrations of the standards. For AAPH, 90% and 110% of the chosen AAPH concentration were tested and three different GA standard concentrations (10, 15 and 20 mg/L) were measured. From the mean value of the corresponding AUC values, the response was also calculated as %. The same standard concentrations were also used to test the effects of changing the fluorescein concentrations between 90% and 110% of the chosen value.

## 3. Results and Discussion

### 3.1. Methodology Validation

Prior to measurements, the whole FIA lab system was thoroughly purged with freshly degassed ethanol and only freshly degassed water was used for preparation of samples and solutions. This turned out to be necessary in order to avoid the formation of air bubbles in the FIA system and to optimize the performance. For the FC assay, the dynamic range was determined to lie between 2 and 60 mg/L of GA, and a correlation coefficient of 0.9996 was obtained for a calibration curve in this concentration range. Measurements of the antioxidative response of GA standard solutions in the range 2 to 8 mg/L gave a LoD of 1.6 mg/L GA and a LoQ of 3.2 mg/L GA. In addition, when solutions of freeze-dried coffee samples (160 mg/L) were spiked with 30 mg/L GA, a recovery of 93% was calculated. The intraday repeatability was 0.9% RSD and the interday reproducibility (over three days) was 2% RSD (Table 2). Compared to the other two assays, this assay showed the highest reproducibility, the lowest sensitivity, and the highest dynamic range (Table 2).

**Table 2:** Validation of analytical results for the FC, ABTS and ORAC assay.

Validation criterion	FC	ABTS	ORAC
Intraday RSD (%)	0.9 at 30 mg/L GA	0.4 at 3 mg/L GA	4.7 at 10 mg/L GA
Interday RSD (%)	2.1 at 30 mg/L GA	4.4 at 3 mg/L GA	8.2 at 10 mg/L GA
LoD (mg/L GA)	1.6	0.12	0.43
LoQ (mg/L GA)	3.2	0.4	2.1
Linearity range (mg/L GA)	2–60	0.5–5	5–25
*R*^2^	0.9996	0.9987	0.9993
Recovery (%)	93 (of 30 mg/L GA)	89 (of 2 mg/L GA)	78 (of 20 mg/L GA)

All validation measurements were performed with gallic acid (GA). LoD = limit of detection, LoQ = limit of quantification, RSD = relative standard deviation.

The dynamic range for the ABTS assay was 0.5 to 5 mg/L, considerably lower than with the FC assay, and the calibration curve for these concentrations showed a correlation coefficient of 0.9987 (Table 2). Here the maximum value of 5 mg/L could not be exceeded since that resulted in zero absorbance of ABTS^•+^. The LoD was determined as 0.12 mg/L GA and LoQ as 0.4 mg/L GA. Interday reproducibility was measured over three days with a deviation of 4.4% RSD and the intraday repeatability was 0.4% RSD. A recovery of 89% was determined for freeze-dried coffee samples (40 mg/L) spiked with 2 mg/L of GA (Table 2). Inhibition percentage was used for quantification, as the recovery values for absorbance drop were considerably lower. For the ABTS assay, repeatability was excellent and reproducibility was acceptable, although better values (1.3% to 1.95% RSD) have been reported for a FIA instead of a SIA setup [37,39]. On testing the stability of ABTS^•+^ for an experimental time of 8 h, the slope of degradation (AU in 10 h) was found to be −0.026 with an *R*^2^ of 0.82. The variation of the results was small with a %RSD of 0.6 (*n* = 8). A similar decrease in slope (−0.023 AU in 10 h) was observed over 13 h with a *R*^2^ of 0.71 and a variation of 0.8% RSD (*n* = 13). Variations in the initial ABTS^•+^ concentration were studied and the effect on the deviation in measured inhibition percentage was estimated. Here with a higher absorbance of 0.88 AU, the deviation was −15.8% and for the lower value of 0.60 AU it was +18.2% compared to the values obtained for 0.7 AU. From these values the maximum allowable deviation of the initial ABTS^•+^ absorbance was calculated as ±0.025 absorption units (AU), which would be within a 3% error margin.

For the ORAC assay, the linearity range was between 5 and 25 mg/L GA and a calibration curve within that range produced a correlation coefficient of 0.9993 (Table 2); the LoD was 0.43 mg/L GA and LoQ 2.1 mg/L GA. The intraday repeatability was calculated as 4.7% RSD and the interday reproducibility over five days was 8.2% RSD. Recovery of 78% was determined for freeze-dried coffee solutions (2000 mg/L) spiked with 15 mg/L of GA (Table 2). The reproducibility of the ORAC measurements, which were made in series with SIA analyses, was in the range of other studies. The literature reports values for the intermediate precision relative standard deviations (RSD_int_) of 0.7 to 13% and reproducibility relative standard deviations (RSD_R_) of 4.4 to 13.8% [36,40,41], which were achieved by parallel measurements using a fluorescence plate reader. Hence, the reproducibility of the assay was still acceptable despite the rather long reaction times. The stability of AAPH was tested hourly by injecting a 20 mg/L GA standard. For 30 h and 30 injections the RSD was 3%, for 40 h and 40 injections it was 4.3%. Also initial concentrations of AAPH as well as fluorescein were varied to assess the importance of precise initial concentrations. Relative concentrations of 90% and 110% were tested for the two substances, which resulted in corresponding responses of 89% and 111% when varying fluorescein, and 107% and 86% when varying AAPH. The main drawback of this assay is the amount of time required to measure samples, but the validation procedure gave a time window of ≥40 h during which the assay conditions remained stable. A major factor affecting the performance of the assay is the stability of radical generator AAPH, which is temperature sensitive [42]. As we needed a valid time period of 40 h, it was important to ensure the same AAPH quality for this time range. Therefore, it was stored in ice water and was purged before each run to have fresh AAPH solution for radical generation. Despite the long reaction time of 1 h, results with acceptable reproducibility were obtained.

### 3.2. Effects of Coffee Roasting Conditions on Antioxidant Contents

**Figure 2:**
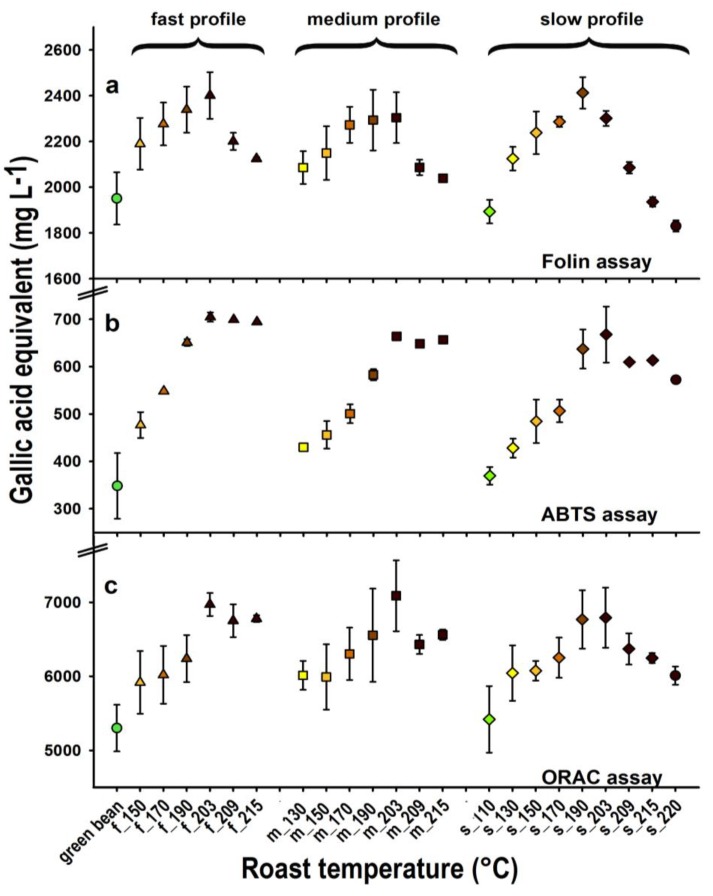
Antioxidant capacity (as gallic acid equivalents, GAE, mean ± SD) of green and roasted coffee measured using (**a**) the Folin-Ciocalteu, (**b**) the ABTS, and (**c**) the ORAC assays. Three roast profiles (fast, f; medium, m and slow, s) were conducted and coffee beans were removed at the given temperatures. The values are depicted with colors from green to brown with increasing roast degree; six roast degrees for the fast profile, seven for the medium profile, and nine for the slow profile were roasted (roast replicate *n* = 2, except for the higher roast temperatures 209, 215, 220 °C, which were only roasted once (brewing replicates *n* = 3).

The results from the assays of the extracts from coffee samples roasted to various stages are presented in Figure 2. Large differences in the antioxidant values were obtained from the different methods, which increases in the order ABTS < FC < ORAC, when compared to GA as reference standard; the mean values for the green coffee, for example, were 373, 1885, and 5083 mg/L GA equivalents, respectively. Clearly GA and the antioxidant components of green coffee behave differently with respect to the three assays, even though phenolic compounds (the CGAs) are the major antioxidants in green coffees.

The water content of the green beans was 10.9%, but this decreased with increasing roast temperature/roasting time within each roasting profile (Table 1). However, when comparing the data between roasting profiles, the color, mass loss and water (humidity) contents were comparable at similar sampling temperatures (130 to 209 °C). The trends with respect to roasting progression were similar for each of the analytical methods, and antioxidant values for all roasted samples were greater than those of the green beans, except the first slightly roasted coffee sample of the slow roast (110 °C). Thus during roasting to a light degree (up to 200 °C), molecules with antioxidant properties are produced in greater amounts than those that are destroyed. Although part of this increase in measured antioxidant capacity can be explained by mass loss during roasting, this loss is insufficient to fully explain the increase (see Table 1). Furthermore, while we attempted to produce samples with consistent extraction efficiency by grinding them with a ball mill, we cannot rule out the possibility that the more voluminous and porous roasted and ground coffee beans resulted in higher extraction efficiency than the harder green coffee beans. Antioxidant values tended to increase with increasing temperature and reached an optimum at around 200 °C [43]. Especially with the FC method, large decreases towards darker roasts were observed. Since the FC method is often considered to be especially sensitive to phenolic compounds, these latter results can be explained by the greater degradation of CGAs at higher temperatures. The effect is strongest with the slow roast, where the energy input is maintained over the longest time span, and the change in magnitude of the antioxidant activity is less pronounced with the ABTS and ORAC assay, which also indicates that these methods have different sensitivities to other types of antioxidants in the roasted coffee [20].

Finally, with each assay there was little influence of the speed of roasting on the antioxidant values observed for each temperature, with the exception of the samples heated to the highest temperature for medium and slow roasting times in the FC assay, where the decrease is increasing with roast length. If, as suggested in the previous paragraph, these lower results with the FC assay correspond to increased destruction of the CGAs, it would appear that the speed of roasting could be an important parameter for the preservation of the CGA component in roasted coffee samples. This effect of speed on the preservation of antioxidant activity is similar with the ABTS assay, as also here the highest increase is measured with the fast roast profile.

### 3.3. Significance of the Numbers Produced by the Different Methods

The FC assay [34] is often considered to be a measure of “total phenolic content”, and since the CGAs represent the main polyphenols in coffee [14], it is tempting to describe the FC values for green coffee as approximating to the CGA concentration. However, this cannot be the situation for the roasted coffee samples, since roasting results in appreciable increases in antioxidant values even though it is known that progressive degradation of CGAs occurs during the roasting process [17,18]. Furthermore, as reported by Prior *et al.* [22], the FC reagent is not specific for phenolic compounds and it can also be reduced by many non-phenolic compounds, e.g., melanoidins [19]; thus it may be more accurately described as measuring the total reducing capacity of a sample [23]. The high pH (pH 10) used in the FC assay results in deprotonation of one phenolic –OH group, a process important for the autoxidation reaction of polyphenols. Thus phenoxy or semiquinone radicals are readily formed at high pH, and it seems likely that such radicals are the reactive compounds in this assay. However, although semiquinone radicals tend to be reasonably stable, at high pH, secondary reactions, such as dimerization, degradation and further oxidation reactions involving generation of some –OH groups can lead to higher activity than would be expected from oxidation of simple catechol groups [44]. Furthermore, cyclic voltammetry studies have shown that oxidation of 5-CQA is not reversible at high pH [45]. Also, phenoxy radicals are often quite reactive and may thus react with other components in the complex coffee matrix. Thus there are potentially reactions occurring in the assay that could lead to either over- or underestimation of the actual phenolic contents of the coffee brews.

In the ABTS assay, the test sample is added after the ABTS^•+^ has been generated, and the amount of radical remaining after a certain reaction time is determined. As with the FC assay, the ABTS assay is not specific for any particular type of antioxidant, since any compound with a redox potential lower than that of ABTS^•+^ (E = 0.68 V) may react with the radical [46].

However, the competitive mechanism of the ORAC assay provides a fundamentally different approach to antioxidant measurement compared to the FC and ABTS assays. By integrating the response over time, this assay includes the reducing effects of secondary components in the sample for which there may be an appreciable lag time before they show a response. Nevertheless, the long time required for the assay represents a major drawback for the routine use of the ORAC assay, which also has technical limitations related to the formation of the peroxyl radicals. For example, radical forming compounds such as AAPH are sensitive to temperature and can undergo spontaneous decay. In air-saturated solutions, it is thought that the AAPH radical reacts rapidly with O_2_ to give a more stable radical, and the loss of fluorescence is then an indication of the extent of damage resulting from reaction with the radicals. However, a big advantage of this assay is the combined quantification of initial reaction rate as well as the total amount of inhibition, which includes slow-reacting or secondary antioxidant products [36]. This combination makes it a useful assay to determine antioxidant capacities of samples with a complex matrix and mixtures of antioxidant active compounds such as coffee. A lag phase could be the consequence of a reaction between fluorescein radicals and antioxidant compounds which leads to regeneration of the original fluorescein [38]. A considerable factor affecting the ORAC assay is the presence of metal ions, which can be responsible for a possible underestimation of the numbers generated [47].

Although there has been increasing use in recent years of measures of antioxidant capacities of food products as markers of their contributions to healthy diets [32], the wide disparity in the numbers obtained by different methods in the present work shows that caution should be exercised in their interpretation. The more than 10-fold range in values for the antioxidant content (expressed as GA equivalents) of green (unroasted) coffee in the present work is a reflection of the different chemical processes involved in the three assays used. If reactions of GA, which was used as a reference standard throughout, were truly representative of phenol chemistry, then the diverse numbers could illustrate variability in the relative sensitivity of the various methods to phenolic and non-phenolic antioxidants. However, the results with green coffee suggest that there are also appreciable differences in the chemical behavior of GA and the CGAs (the major antioxidants in green coffee) under the conditions of the different assays. As a consequence, we compared the responses of GA and 5-CQA in the three assays. Considerable differences were observed, and on a molar basis GA was more reactive than 5-CQA in the ABTS assay, but less reactive in the FC and the ORAC assays. When the antioxidant capacity of green coffee was recalculated as CGA equivalents (CGAE), mean values of 3874, 3080 and 2671 mg/L were obtained for the ABTS, FC and ORAC assays, respectively. These are much closer than the corresponding numbers (373, 1885, and 5083 mg/L) for GA equivalents, and are also consistent with CGAs being the main antioxidants in green coffee.

The differences in the responses of the standard compounds GA and 5-CQA in the ABTS assay are pronounced with GA values being ~five times higher for the same concentration. This is broadly in line with other publications, where GA has also been reported to be around 2.7 to 4.3 times more potent as a reductant than CGA or caffeic acid [18,39,48,49]. However, the analyses of green coffee gave values >10 times higher for CGAE than GAE values. The reaction time was then considered as a possible factor affecting the behavior of GA and 5-CQA in the ABTS assay, since the antioxidant capacity determined for phenolic compounds using this method has been reported to be sensitive to the choice of analytical parameters [50]. However, in time delay experiments with concentrations that give similar responses in the ABTS assay (2 mg/L GA and 15 mg/L 5-CQA), we found that both phenols produced similar absorption intensity curves (data not shown). Furthermore, Tian and Schaich [51] have reported that 5-CQA reacts essentially instantaneously with ABTS^•+^, so the cause of the different behavior of GA and 5-CQA in this assay is probably chemical rather than physical.

The differences in the responses of GA and 5-CQA in the FC and ORAC assays were less pronounced, than in the ABTS assay, but not insignificant. In the FC assay, the value for GA was ~0.8 times that of 5-CQA, which is similar to the result reported by Chun and Kim [52], although on the basis of the number of hydroxyl groups, the antioxidant capacity of GA should be higher [18,53]. The number of electrons involved in oxidation increased for CGA with increasing pH, which could also be an explanation for the observed higher antioxidant potential of 5-CQA in the present study [45]. With the ORAC assay, the response of 5-CQA was 2.5 times that of GA, and an even greater ratio (3.5) has been reported by Yeh and Yen [49]. These latter results could be the consequence of secondary reactions which also contribute to the antioxidant capacity determined by this method [53], and in the coffee extracts there could be further complications arising from the presence of transition metal species [48]. It should be noted that the ORAC assay was run for ≥30 min to avoid any underestimation of antioxidant capacity due to slow reacting antioxidants.

Recent studies of coffee brews using high performance size exclusion chromatography coupled to on-line assays have suggested that CGAs and melanoidins (the components responsible for the largest contributions to the antioxidant capacity of coffee brews) produce different responses in the FC and ABTS methods [18]. In that work it was concluded that the ABTS assay was relatively more sensitive towards melanoidins than the FC assay, which is also supported by the present results. Furthermore, the contributions to the antioxidant capacity derived from CGAs decreased with a higher roasting degree, whilst there was only a minor increase in the contribution from melanoidins. For the coffee samples roasted to a light roast, only a small amount of degradation of CGAs was observed with the FC assay. However, this was accompanied by a larger increase in the melanoidin contribution, a result that suggests that melanoidins produced in the early stage of roasting have higher antioxidant potential than those produced at later stages. This hypothesis will be tested in future experiments, as well as the extent of contributions from other components in the coffee brews.

Although this research shows the progressive formation of antioxidant molecules during the coffee roasting process, it also highlights a fundamental problem in interpreting results from antioxidant assays. Different molecules have different relative responses in different assays, and even phenols can have widely different relative responses in commonly-used assays. Because of this, the choice of reference standard can have a major influence on the results, as illustrated by the numbers from the various assays expressed as GA or CGA equivalents. Problems relating to the selection of reference standards in antioxidant activity assessment have been discussed in detail by Nenadis *et al.* [54]. However, apart from recommending that uric and ascorbic acids should not be used for this purpose, they concluded that there was no single ideal reference standard, whilst lending some support to the use of Trolox. Also, in the six assays considered in that paper, the GA/Trolox response ratio varied between 0.52 and 3.76. Furthermore, if the antioxidant values for green coffee beans in the present work are expressed relative to Trolox using the relative activities of GA and Trolox reported by Nenadis *et al.* [54], values of 1200, 7090, and 5340 mg/L are obtained for the ABTS, FC and ORAC assays, respectively.

In addition to these analytical problems, it is also unlikely that all antioxidants in foods behave in the same way after consumption. Thus, whereas some may be taken up intact during food digestion, others may be altered, and yet others may be unavailable, e.g. liposolubility. Therefore, questions must be raised as to the relevance of analyses such as those presented here to the health of consumers, and until we have more information, caution should be exercised in interpreting antioxidant values beyond simple shelf-life prediction. Furthermore, even the use of such antioxidant assays for shelf-life prediction requires calibration with actual storage-induced changes, possibly on a product-by-product basis.

## 4. Conclusions

The results from this work show clearly the progressive generation of antioxidant molecules during the roasting of coffee, and that this occurs more rapidly than any degradation of the initial antioxidant capacity during the early stages of the roasting process. After an optimum value at around 200 °C, the antioxidant activity decreased due to degradation processes. Similar trends were observed with three different types of assay that used different types of reagents, and hence putatively had different responses to antioxidant molecules in the coffee; the active reagents were first a metal complex, second a stable radical, and third a radical with moderate stability. However, these three analytical methods produced widely different numbers relative to the GA standard, although much more similar numbers were obtained when the results were expressed as CGA equivalents. Reproducibility of replicate samples was good, and each method showed a consistent set of trends in antioxidant values with roasting conditions. Thus there are differences in the chemical nature of the components measured with the individual methods, a conclusion which indicates that the concept of a single antioxidant number is meaningless, especially in the context of the “healthiness” of a food product. Profiles of antioxidant properties from complementary methods are potentially more meaningful, but the extent to which differences in biological behavior are reflected by the results from the various assays is still unknown. However, although the analytical results described in the present paper give a representation of various aspects of the antioxidative capacity in coffee; it represents just a first step towards developing knowledge about the health effects of the beverage. Nevertheless, it demonstrates the necessity of using a combination of assays for the determination of antioxidant properties of food products. The methods presented here are sufficiently simple that they can be applied routinely in food quality analysis, although at the present time we still do not have a definitive interpretation of the meaning of the numbers generated.
